# Data on immunoglobulin G antibodies induced by immunization of mice with globoside carrying very long-chain fatty acids

**DOI:** 10.1016/j.dib.2018.05.014

**Published:** 2018-05-10

**Authors:** Tetsuya Okuda

**Affiliations:** Bio-Design Research Group, Bioproduction Research Institute, National Institute of Advanced Industrial Science and Technology (AIST), Central 6, 1-1-1 Higashi, Tsukuba, Ibaraki 305-8566, Japan

## Abstract

The data presented in this article are related to the research article entitled “Generation of anti-oligosaccharide antibodies that recognize mammalian glycoproteins by immunization with a novel artificial glycosphingolipid” (Okuda and Fukui, 2018) [1]. This article describes the immunogenicity of a mammalian glycosphingolipid (globoside) carrying very long-chain fatty acids. Analysis of serum antibody titer by ELISA showed that this globoside had a strong immunogenicity in mice and could immediately induce production of anti-globoside IgGs. Isolated an IgG3 (κ) monoclonal antibody (mAb PA4.2) from the immunized mouse showed high specificity and reactivity against globoside. These data provide a novel antigen design method useful for obtaining IgG antibodies against glycosphingolipids.

**Specifications Table**

**Table:** 

Subject area	*Immunology*
More specific subject area	*Antibody development, Glycosphingolipid, Globoside*
Type of data	*Serum antibody titer, Biochemical properties of monoclonal antibodies*
How data was acquired	*ELISA using a TMB substrate (1-Step Ultra TMB-ELISA Substrate Solution, Pierce, Rockford, IL) and a micro plate reader (SpectraMax Paradigm Multi-Mode Microplate Reader, Molecular Devices, San Jose, CA).*
	*TLC-immunostaining using a horseradish peroxidase (HRP)-labeled secondary antibody and a HRP substrate (Immunostain HRP-1000, Konica Minolta Medical & Graphic, Inc., Tokyo, Japan).*
Data format	*Raw data for ELISA and TLC-immunostaining*
Experimental factors	*Serum of mice immunized with human erythrocyte-derived globoside, and culture supernatants of hybridoma cells producing anti-globoside antibodies.*
Experimental features	*Mice were immunized with human erythrocyte-derived globoside, and antibody titers against globoside were evaluated by ELISA. Hybridoma cells were generated from the immunized mice, and two clones (PA4.2 and PA5) that produced anti-globoside monoclonal antibodies were isolated. The properties of these antibodies were evaluated with ELISA and TLC-immunostaining.*
Data source location	*Bioproduction Research Institute, National Institute of Advanced Industrial Science and Technology (AIST), Central 6, Tsukuba*
Data accessibility	*The data are available with this article.*

**Value of the data**

• The data demonstrate that the glycosphingolipid carrying very long-chain fatty acids shows strong immunogenicity in mice.
• The data and protocols providing here support other researchers to develop anti-glycosphingolipids antibodies.
• The anti-globoside IgG3 antibody was established for the first time. It will be a valuable tool for researchers from related fields.

## 1 Data

**Fig. 1:**
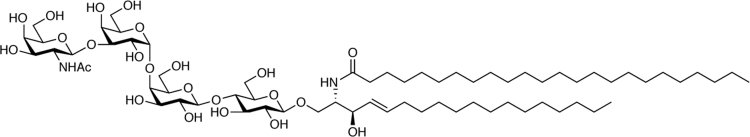
Representative structure of human erythrocyte-derived globoside. Globoside derived from human erythrocyte predominantly contains C24 fatty acids [2]. This figure illustrates globoside containing C24:0 fatty acid.

**Fig. 2:**
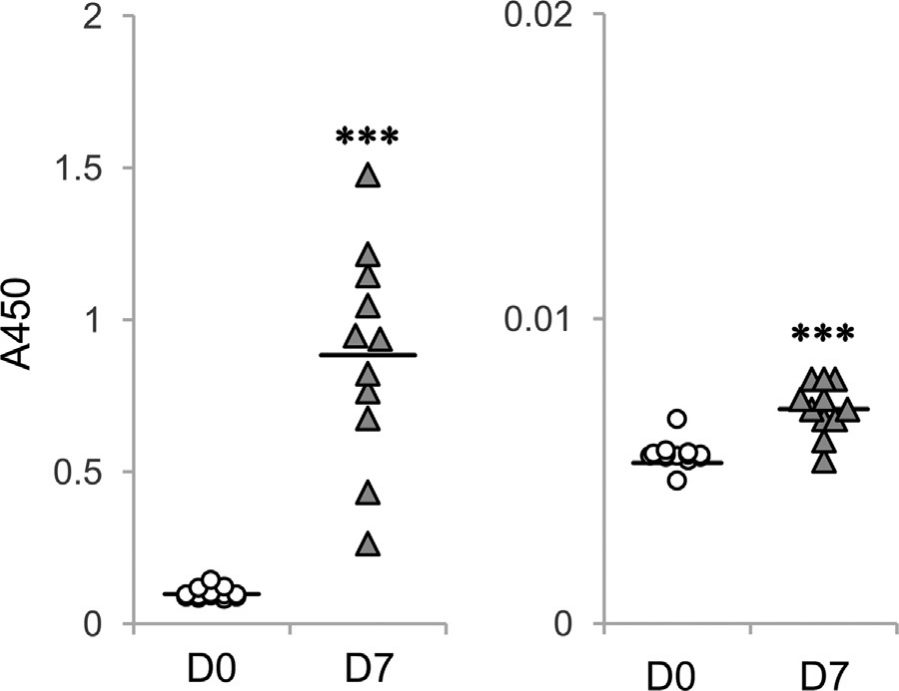
Serum antibody titers in globoside-immunized mice. Mice were immunized with globoside by a liposome method [3], and sera were collected at 7 days (D7) after a booster immunization. Left panel, anti-globoside IgM titer. Right panel, anti-globoside IgG titer. D0, sera from non-treated mice. ***P<0.001 D0 vs. D7 (*n*=11).

The human erythrocyte-derived globoside has known predominantly carrying very long-chain fatty acids in their fatty acid portion [2]. The representative structure is shown in Fig. 1. We immunized mice with this globoside by a liposome immunization method [3]. Sera were collected at 7 days after a booster immunization, and anti-globoside IgM or IgG antibody titers in the sera were evaluated by ELISA (Fig. 2). The data indicate that immunization with this globoside immediately induces anti-globoside IgM and IgG production in these-immunized mice.

**Fig. 3:**
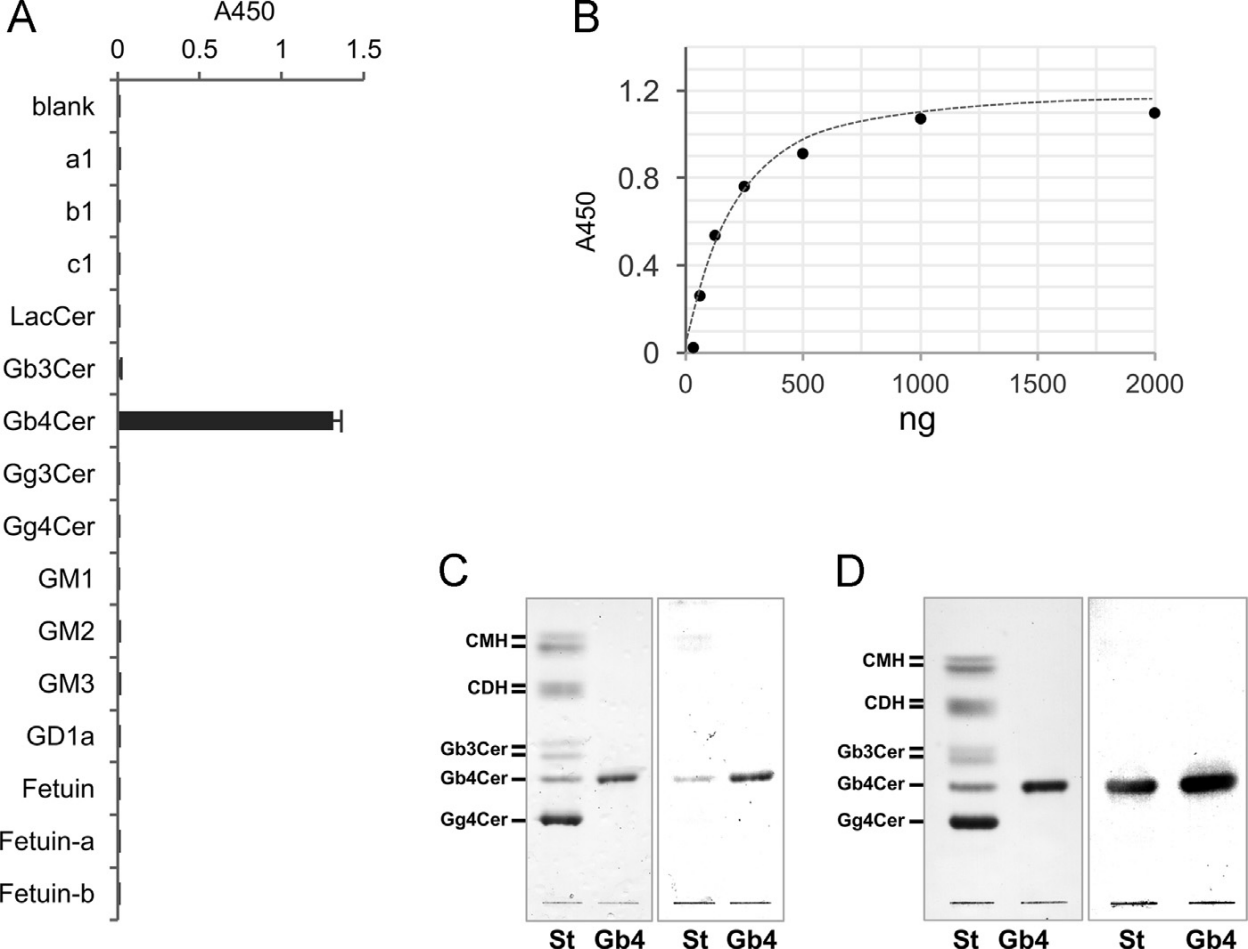
Characterization of monoclonal antibody PA4.2. (A) Reactivity of mAb PA4.2 to several glycoconjugates evaluated by ELISA. a1, b1 and c1 are artificial glycosphingolipids containing 6′-sialy LacNAc, 3′-sialy LacNAc, and LacNAc, respectively. These chemical structures were described in Ref. [1]. LacCer, Galβ1,4Glcβ1Ceramide; Gb3Cer, Galα1,4Galβ1,4Glcβ1Ceramide; Gb4Cer, globoside/GalNAcβ1,3Galα1,4Galβ1,4Glcβ1Ceramide; Gg3Cer, GalNAcβ1,4Galβ1,4Glcβ1Ceramide; Gg4Cer, Galβ1,3GalNAcβ1,4Galβ1,4Glcβ1Ceramide; GM1, Galβ1,3GalNAcβ1,4(Neu5Acα2,3)Galβ1,4Glcβ1Ceramide; GM2, GalNAcβ1,4(Neu5Acα2,3)Galβ1,4Glcβ1Ceramide; GM3, Neu5Acα2,3Galβ1,4Glcβ1Ceramide; GD1a, Neu5Acα2,3Galβ1,3GalNAcβ1,4(Neu5Acα2,3)Galβ1,4Glcβ1Ceramide; Fetuin-a, desialylated Fetuin; Fetuin-b, α2,3-sialidase-treated Fetuin. (B) Reactivity of mAb PA4.2 to several amounts of globoside evaluated by ELISA. (C) TLC-immunostaining of globoside using PA4.2. Left panel, orcinol-H_2_SO_4_ staining. Right panel, immunostaining with PA4.2. St, standard glycosphingolipids mixture; Gb4, globoside. CMH, ceramide monohexoside; CDH, ceramide dihexoside. (D) TLC-immunostaining of globoside using PA5. Left panel, orcinol-H_2_SO_4_ staining. Right panel, immunostaining with PA5.

To develop monoclonal anti-globoside antibodies, splenocytes were obtained from a mouse after four times booster immunizations, and were fused with Sp2/0-Ag14 myeloma cells and cultured in a 96-well plate at a density of 1 colony/well. Anti-globoside antibody titers in the culture supernatants of individual clones were screened by ELISA, and clones with high antibody titer to globoside were selected as positive clones. At this stage, the proportion of the strong positive clones was 7.5%. Hybridoma clones, PA4.2 producing IgG3 (κ), and PA5 producing IgM (κ) were isolated and found to have high specificity and reactivity to globoside (Fig. 3). Although there have been reports on the development of several anti-globoside antibodies [4], this study is the first report on the generation of the monoclonal anti-globoside IgG. The specificity of mAb PA4.2 to antigen was confirmed by ELISA (Fig. 3A) and was found to strictly recognize globoside. PA4.2 has very high affinity to globoside and is capable of quantitatively detecting globoside in amounts down to 10^−8^ g (Fig. 3B). The specificity and reactivity of mAb PA5 have already been reported [4]. The PA4.2 and PA5 showed the same degree of specificity in detecting globoside in TLC-immunostaining as in ELISA (Fig. 3C and D).

## 2 Experimental design, materials and methods

### 2.1 Immunization and preparation of serum

The C3H/HeN mouse strain (CREA Japan, Tokyo) was used in this study. A liposome immunization method [3] was used for immunization of globoside. In brief, 100 μg of globosides were mixed with 10 μg of lipid A, 0.5 μmol of cholesterol and 0.5 μmol dipalmitoylphosphatidylcholine. The mixture was dissolved in PBS and used as an immunogen. The animals were first subcutaneously immunized, followed by intraperitoneal immunization two weeks after the first immunization. Serum was prepared from tail vein blood of mice at seven days after the second immunization. The Committee for the Experiments involving Animals of the National Institute of Advanced Industrial Science and Technology (AIST) approved all animal experiments.

### 2.2 Enzyme-linked immunosorbent assay (ELISA)

The ELISA analyses were performed as described previously [4]. In brief, glycosphingolipids and glycoproteins were applied onto a 96-well microtiter plate and were incubated overnight. After washing twice with PBS, blocking buffer (1% bovine serum albumin in PBS) was added into each well and incubated for 15 min at room temperature, followed by the addition of diluted serum (1:100) or hybridoma culture supernatant (1:1). After 3 hours incubation at room temperature, the wells were washed by 0.1% Tween 20 in PBS and HRP-linked secondary antibody (anti-IgM or anti-IgG) was added. An HRP substrate (1-Step Ultra TMB-ELISA Substrate; Pierce, Rockford, IL) was used to detect antibody binding, and the results were measured as absorbance at 450 nm.

### 2.3 Hybridoma generation

The immunized mice described in the Immunization and preparation of serum section was immunized an additional three times at two-week intervals. Three days after the last immunization, splenocytes were collected from mice and were fused with mouse Sp2/0-Ag14 myeloma cells (RIKEN CELL BANK, Tsukuba, Japan). Hybridomas were selected in HAT selection medium; RPMI-1640 containing 10% FCS, 0.1 mM sodium hypoxanthine, 0.4 µM aminopterin, 16 µM thymidine, 10 µg/ml gentamicin, and 5% Briclone (DS Pharma Biomedical, Osaka, Japan). The culture supernatants were evaluated by ELISA and positive clones were selected using the antibody titer as an index.

### 2.4 TLC-immunostaining

TLC immunostaining were performed as described in Ref. [5]. Glycosphingolipids were analyzed on HPTLC plates (Merck, Darmstadt, Germany) with a solvent system consisting of chloroform/methanol/water (60:35:8, *v/v/v*). Standard glycosphingolipids were visualized by orcinol-H_2_SO_4_. TLC-immunostaining was performed using hybridoma supernatants (1:2). Antibody binding was detected using an ABC kit (Vector Laboratories, Burlingame, CA) and Immunostain HRP-1000 (Konica Minolta Medical & Graphic, Inc., Tokyo, Japan).
